# Effects of anti-epileptic drugs on spreading depolarization-induced epileptiform activity in mouse hippocampal slices

**DOI:** 10.1038/s41598-017-12346-y

**Published:** 2017-09-19

**Authors:** Ching-Huei Lin, Shih-Pin Hsu, Ting-Chun Cheng, Chin-Wei Huang, Yao-Chang Chiang, I-Han Hsiao, Ming-Hsueh Lee, Mei-Lin Shen, Dong Chuan Wu, Ning Zhou

**Affiliations:** 1 Graduate Institute of Biomedical Sciences, China Medical University, Taichung, Taiwan; 2 Translational Medicine Research Center, China Medical University Hospital, Taichung, Taiwan; 3 Department of Neurology, E-DA Hospital, I-Shou University, Kaohsiung, Taiwan; 4 Department of Neurology, National Cheng Kung University Hospital, College of Medicine, National Cheng Kung University, Tainan, Taiwan; 5 Center for Drug Abuse and Addiction, China Medical University Hospital, China Medical University, Taichung, Taiwan; 6 Department of Nursing, Division of Basic Medical Sciences, Chang Gung University of Science and Technology, Chia-Yi, Taiwan; 7 Department of Neurosurgery, China Medical University Hospital, Taichung, Taiwan; 8 Division of Neurosurgery, Department of Surgery, Chang Gung Memorial Hospital, Chia-Yi, Taiwan

## Abstract

Epilepsy and spreading depolarization (SD) are both episodic brain disorders and often exist together in the same individual. In CA1 pyramidal neurons of mouse hippocampal slices, induction of SD evoked epileptiform activities, including the ictal-like bursts, which occurred during the repolarizing phase of SD, and the subsequent generation of paroxysmal depolarization shifts (PDSs), which are characterized by mild depolarization plateau with overriding spikes. The duration of the ictal-like activity was correlated with both the recovery time and the depolarization potential of SD, whereas the parameters of PDSs were not significantly correlated with the parameters of SD. Moreover, we systematically evaluated the effects of multiple anti-epileptic drugs (AEDs) on SD-induced epileptiform activity. Among the drugs that are known to inhibit voltage-gated sodium channels, carbamazepine, phenytoin, valproate, lamotrigine, and zonisamide reduced the frequency of PDSs and the overriding firing bursts in 20–25 min after the induction of SD. The GABA uptake inhibitor tiagabine exhibited moderate effects and partially limited the incidence of PDSs after SD. AEDs including gabapentin, levetiracetam, ethosuximide, felbamate, and vigabatrin, had no significant effect on SD-induced epileptic activity. Taken together, these results demonstrate the effects of AEDs on SD and the related epileptiform activity at the cellular level.

## Introduction

Spreading depolarization (SD, also called spreading depression) is a pathophysiological phenomenon that occurs under many neurological conditions, such as traumatic brain injury (TBI), aneurysmal subarachnoid hemorrhage (aSAH), intracerebral hemorrhage, and malignant cerebral infarction^1,2^. SD is characterized by profound depolarization of neurons and glia, which is accompanied by massive ion exchange across plasma membranes of the affected cells^3,4^. These electrical and ionic changes cause a disturbance in cell metabolism and might lead to cell death in metabolically compromised brain tissue^5^. Interestingly, SD is highly associated with the development of epilepsy in patients with aSAH^6^. In rodent and human brain slices, SD could trigger epileptiform responses that are characterized by ictal-like discharges on the tailing end of the extracellular potential shift of SD^7,8^. After the membrane potential recovers from the depolarization resulting from the SD, the neuronal activity transforms into epileptic discharge patterns that are characterized by paroxysmal depolarization shifts (PDSs)^8^.

PDSs are considered to be the manifestation of epileptic interictal spikes at the level of single neurons^9^. A PDS normally consists of a plateau-like depolarization associated with repetitive discharges of the neuron. The sustained depolarization is initiated by large excitatory postsynaptic potentials (EPSPs)^10^. The repetitive spikes are mediated by activation of voltage-gated Na^+^ channels (VGSCs). Activation of voltage-gated, high-threshold Ca^2+^ conductance and persistent Na^+^ conductance further contributes to the enhancement of depolarization. The repolarization phase of PDS is normally followed by hyperpolarization that involves a GABA_A_ receptor-mediated Cl^−^ conductance and Ca^2+^-dependent K^+^ conductance^11^. A better understanding of the pharmacological sensitivity of SD-induced PDSs will have implications for the treatment of neurological conditions and complications that are associated with SD.

Anti-epileptic drugs (AEDs) include more than twenty molecular entities that are marketed worldwide. AEDs are effective by different mechanisms of action, including modulation of voltage-gated Na^+^ channels (VGSCs) and/or voltage-gated Ca^2+^ channels (VGCCs), enhancement of inhibitory synaptic transmission, or inhibition of excitatory neurotransmission^12,13^. Activation of VGSCs is crucial for the generation of high-frequency repetitive discharges and PDSs, which are responsible for the generation of the ictal and interictal states of the seizure^14^. High-voltage activated VGCCs (L-, P/Q-, N- and R-types) are required for presynaptic neurotransmitter release and might modulate neuronal firing patterns, whereas activation of low voltage-activated VGCCs (T-type) are involved in neuronal bursting^15^. Moreover, some AEDs act at least partially by enhancing GABA transmission or inhibiting ionotropic glutamate receptors to modulate synaptic transmission^16,17^. Different types of AEDs are used for the treatment of different classifications of seizures. However, which type of AEDs are most effective in preventing epileptiform activity induced by SD remains unknown.

In the present study, we systematically evaluated the inhibitory effects of AEDs on SD-induced epileptic activity. The effects of a range of existing AEDs, including carbamazepine, phenytoin, valproate, lamotrigine, zonisamide, felbamate, gabapentin, levetiracetam, ethosuximide, tiagabine and vigabatrin, were tested on the PDSs following SD induction in hippocampal CA1 pyramidal neurons of mouse brain slices.

## Results

### SD induction of epileptiform activity

**Figure 1:**
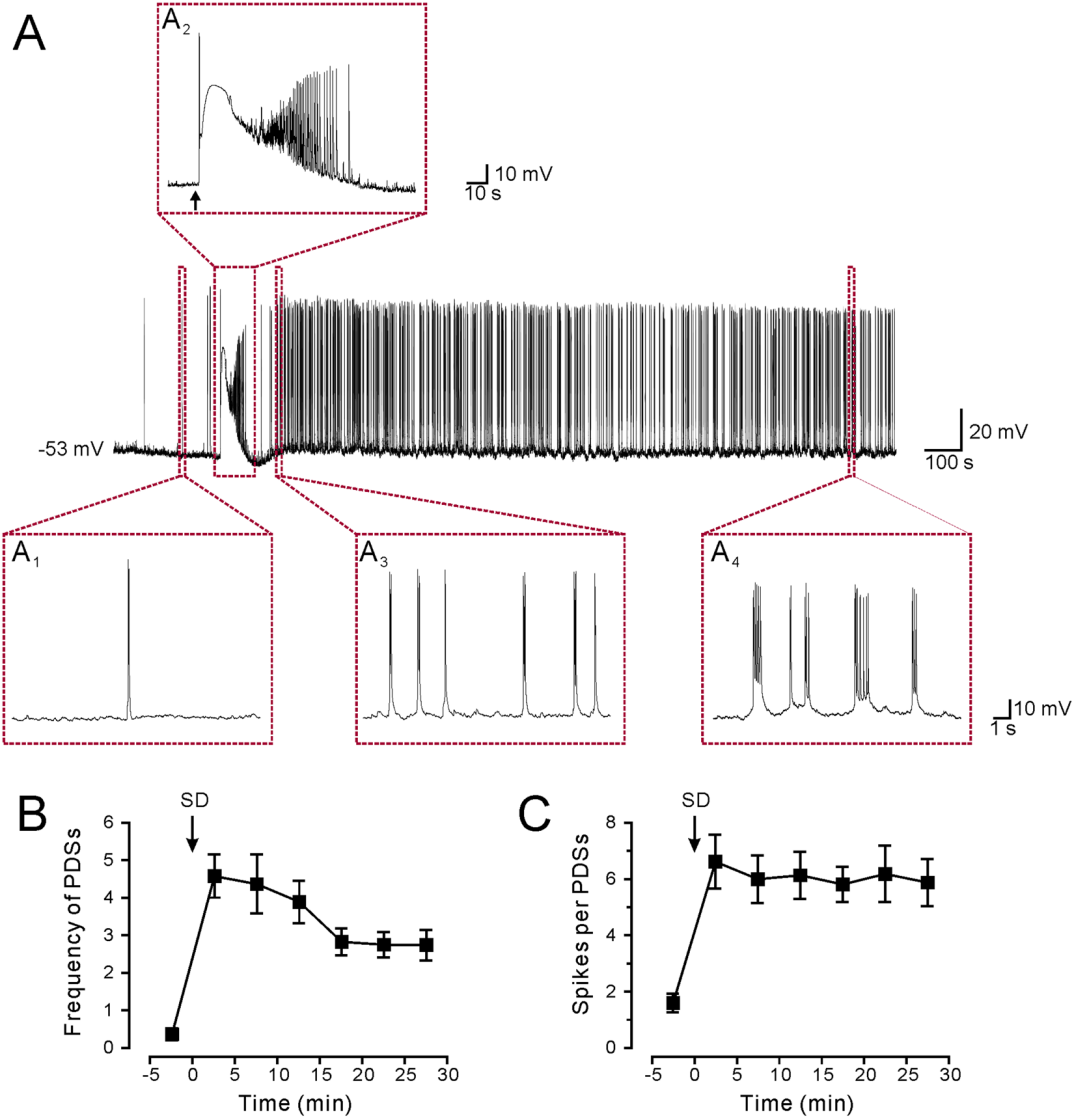
SD induced epileptiform activity in CA1 pyramidal neurons in mouse hippocampal slices. (**A**) A representative trace showing that SD induced PDSs that were sustained for over 30 min in a hippocampal CA1 pyramidal neuron. The changes in the membrane potential over time in the dotted squares are expanded in (A_1_–A_4_). (A_1_) shows that spontaneous firing of action potentials could be recorded before the induction of SD. (A_2_) shows the induction of SD. The application of the high KCl solution from the ejection pipette is indicated by the arrow. The waveform of the SD is characterized by prolonged depolarization for tens of seconds and profound depolarization in the membrane potential. Ictal-like discharges normally appears during the repolarization phase of SD. (A_3_) shows that PDSs instead of action potentials starts to appear after the membrane potential recovered from the depolarization caused by SD. (A_4_) shows further broadening of PDSs and an increased number of spikes during each PDS. (**B**) Quantitative results showing the frequency of PDSs before and after the induction of SD. (**C**) Quantitative results showing the number of spikes during each PDS before and after the induction of SD.

Whole-cell patch clamp recordings were performed in the CA1 pyramidal neurons in mouse hippocampal slices. Under control conditions with physiological levels of extracellular K^+^ and Mg^2+^, prolonged epileptiform activity is rarely observed after SD. Previous studies have shown that SD could evoke long-lasting epileptiform activity in partially disinhibited slices, that is, using 1.25 μM bicuculline to partially block GABA_A_ receptors^8^. This model is, however, not applicable to our study, since AEDs including tiagabine and vigabatrin mainly target on the GABAergic transmission. The network excitability could also be increased by inhibition of certain types of voltage-gated potassium channels with tetraethylammonium (TEA) or Cs^+^. Considering that bath application of potassium blockers will influence the propagating properties of SD^18^, we tried intracellular application of Cs^+^ or TEA, which only modified the excitability of the recorded cell without affecting the propagation of SD or the excitability of the entire circuitry. Intracellular Cs^+^ increased the basal activities of neurons even without the induction of SD, causing a remarkable upshift of the resting membrane potential and massive spontaneous bursting; whereas intracellular TEA did not dramatically affecting the resting membrane potential or causing spontaneous epileptiform activity. Therefore, we tested whether SD could evoke epileptiform activity under a condition of 1.3 mM [Mg^2+^]_o_, 5.0 mM [K^+^]_o_ and internal TEA. In the current-clamp mode (I = 0), the resting membrane potentials of CA1 neurons were scattered between −52 mV to −62 mV in the recorded cells. Spontaneous neuronal firings with typical waveforms of single action potentials could be observed (Fig. 1A_1_). SD was induced by focal KCl ejection from a glass pipette according to a well-established protocol^19,20^. The onset of SD was characterized by a profound depolarization of the membrane potential to −3.6 ± 1.0 mV with an averaged half-width duration of 35.8 ± 1.2 sec (n = 40) (Fig. 1A_2_). Such a depolarization phase represents a typical spreading depolarization as previously reported in human and animal models^20,21^. High frequency spikes at a frequency of 1~5 Hz first appeared during the late repolarization phase of SD^8^, which resembles the ictaform activity observed in spreading convulsions^6^. The ictal-like activity normally started in the late phase of repolarization with a mean duration of 79.2 ± 4.3 sec (n = 40). After the membrane potentials recovered to baseline, another form of epileptiform activity that was characterized by PDSs developed from a pattern of broadened action potentials (Fig. 1A_3_). PDSs were characterized by mild but prolonged depolarization with overriding burst of spikes (Fig. 1A_4_). The occurrence of PDSs normally lasted over 30 min from the onset of SD. In a recording period of 20–25 min after the induction of SD, PDSs occurred at a mean frequency of 2.75 ± 0.34 times/min with an overriding burst of 6.18 ± 1.00 action potentials (Fig. 1B,C). These data indicate that SD induces PDSs that reflect epileptiform activity at the single cell level.

### Relationships among SD characteristics, SD-induced ictal-like activity, and PDSs

**Figure 2:**
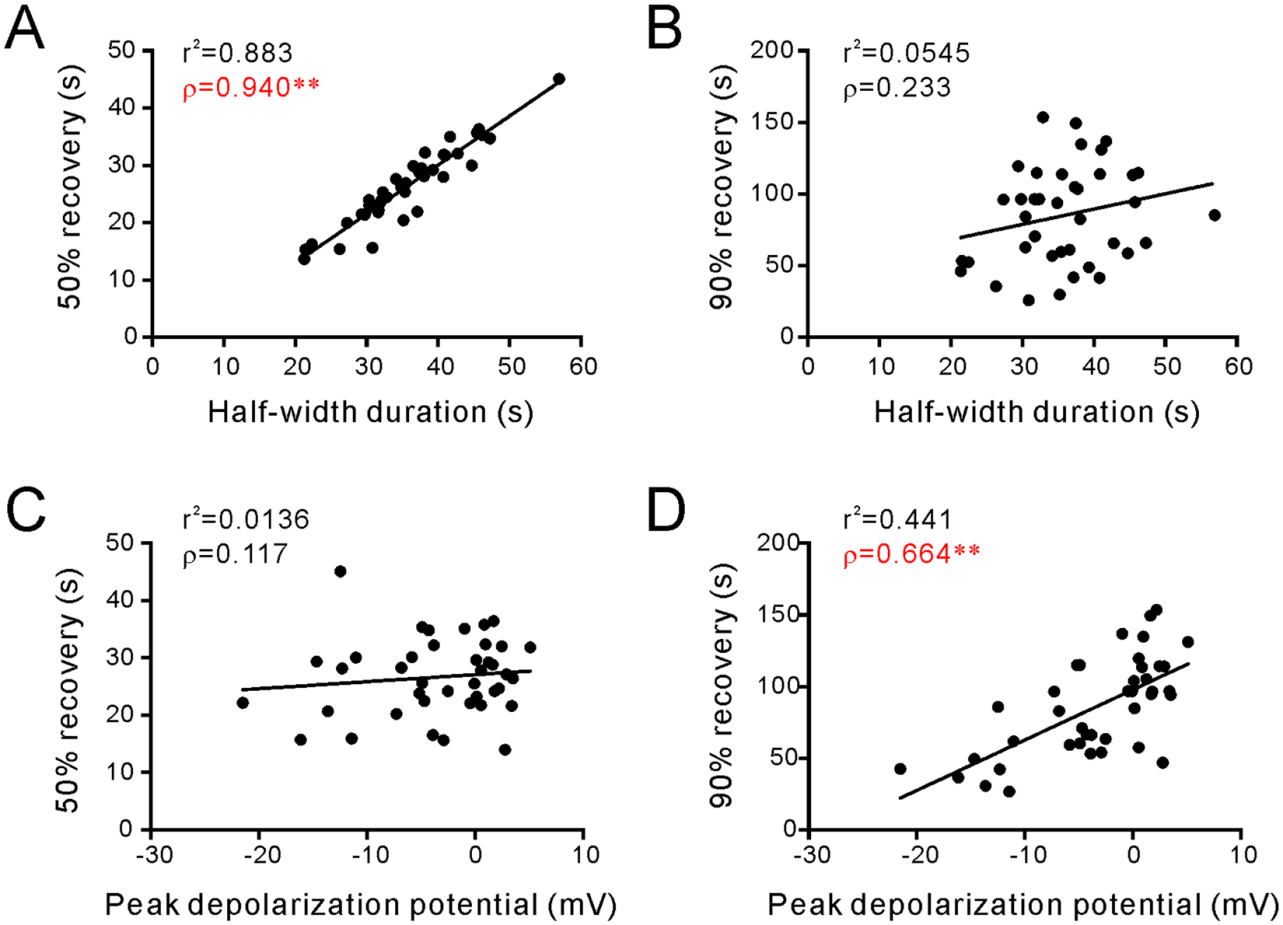
Correlation of parameters of SD. Each point represents a pair of parameters of SD from a recorded neuron (n = 40). (**A** and **B**) The half-width durations of SD are significantly correlated with the τ_50_ (**A**) but not the τ_90_ (**B**) of SD. (**C** and **D**) The peak depolarization potential is not correlated with τ_50_ (**C**) but is rather correlated with the τ_90_ (**D**) of SD. r^2^, R-squared from linear regression analysis; ρ, Pearson’s correlation coefficient; *P < 0.05; **P < 0.01.

As a first step towards a better understanding of the associations between SD and the epileptiform activity, we measured the 50% (τ_50_) and 90% recovery time (τ_90_) of SD, considering that the ictal-like activity normally started in the late repolarization phase of SD. τ_50_ and τ_90_ were determined by the duration from the time of the peak depolarization potential to the time at which the membrane potential reaches the 50% and 90% of full recovery, respectively. τ_90_ was measured instead of the full recovery time because τ_90_ is easier and more reliable to be defined. Our results showed that SD recovered with a mean τ_50_ = 26.5 ± 1.1 sec and a mean _90_ = 85.0 ± 5.4 sec (n = 40). The relationship between τ_50_/τ_90_ and other parameters of SD was assessed by two-tailed Pearson’s correlation analysis. The results show that τ_50_ is highly correlated with the half-width duration of SD (Fig. 2A); interestingly, however, τ_90_ has no significant correlation with the half-width duration (Fig. 2B). Furthermore, the peak depolarization potential was significantly correlated with τ_90_ but not τ_50_ (Fig. 2C,D). These data suggest that the time required for the near complete recovery was highly dependent upon the extent of membrane potential depolarization of the SD, whereas τ_50_ is not directly associated with the depolarization potential.

**Figure 3:**
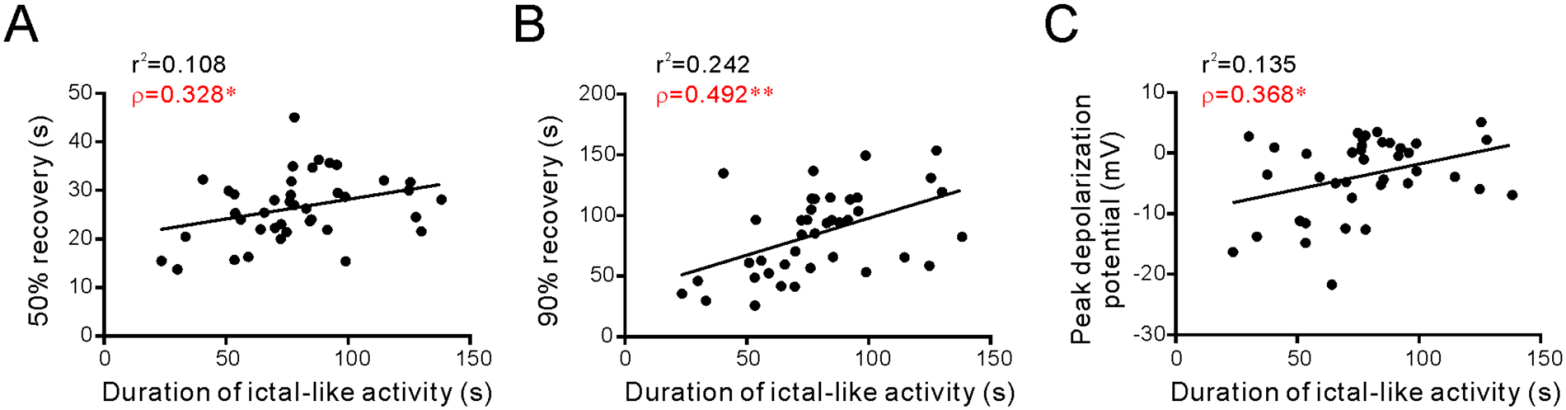
Correlation of ictaform duration and parameters of SD. Each point represents a pair of ictaform duration and a parameter of SD from a recorded neuron (n = 40). The durations of ictal-like activity are significantly correlated with the τ_50_ (**A**), τ_90_ (**B**) and the peak depolarization potential of SD (**C**). r^2^, R-squared from linear regression analysis; ρ, Pearson’s correlation coefficient; *P < 0.05; **P < 0.01.

Considering that the ictal-like activity unusually occurred during the late repolarization phase of SD, it would be interesting to examine its relationships to the SD depolarization potential and recovery time. The results showed that duration of the ictal-like activity was significantly correlated with τ_50_, τ_90_, and the peak depolarization potential of SD (n = 40, Fig. 3). These data suggest that higher extent of membrane depolarization likely facilitates a longer period of ictaform events following SD.

**Figure 4:**
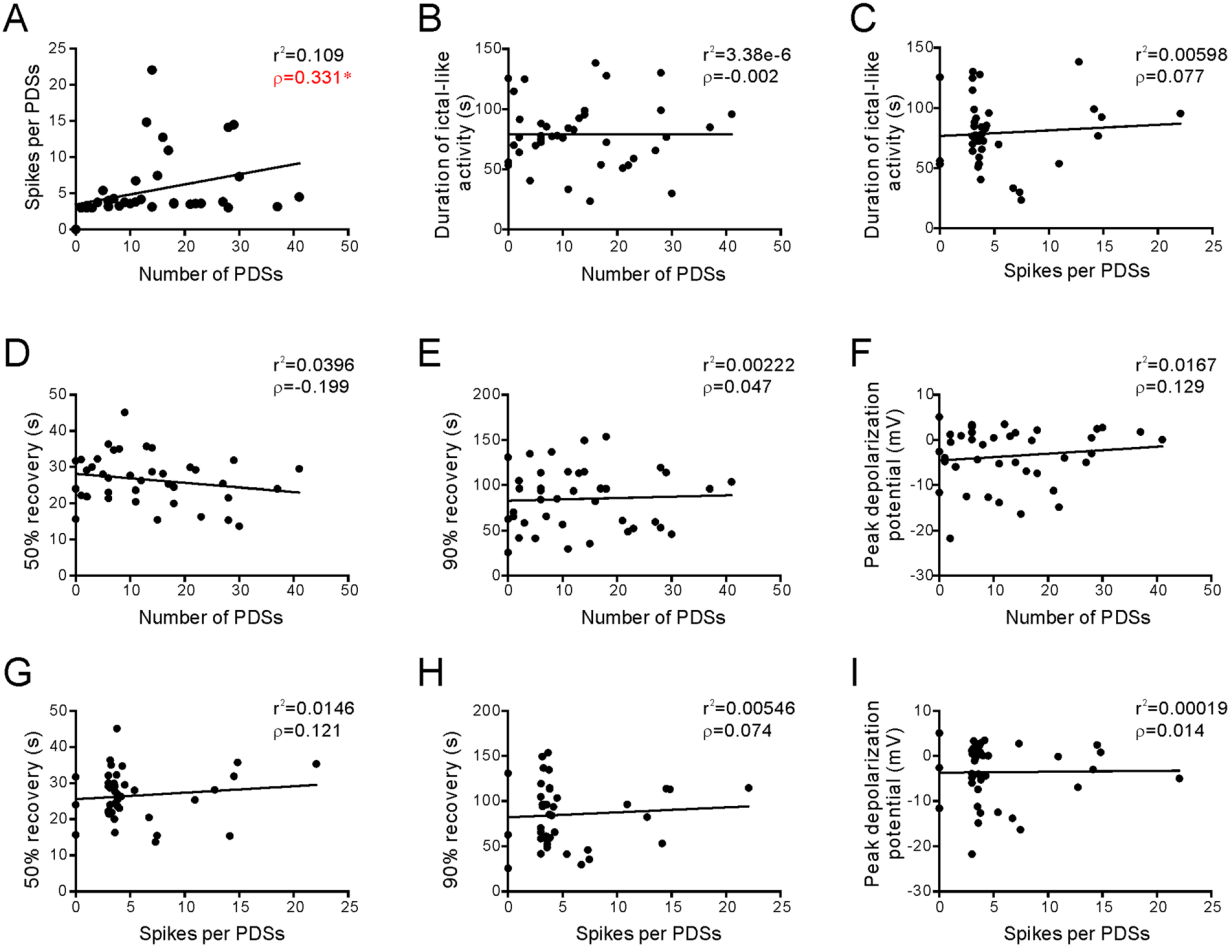
Correlation of PDS parameters, ictaform durations, and parameters of SD. The PDS parameter, the ictaform duration, and the SD parameter from a recorded neuron are graphed in all pairwise combinations (n = 40). (**A**) The number of PDSs (as the total PDSs calculated in the 20-25 min after SD induction) is significantly correlated with the spike numbers during each PDS. (**B** and **C**) Neither the number of PDSs (**B**) or the number of spikes (**C**) is significantly with the duration of ictal-like duration. (**D** and **F**) There is no significant correlation between the number of PDSs and the parameters of SD. (**G** and **I**) There is no significant correlation between the spike number per PDS and the parameters of SD. r^2^, R-squared from linear regression analysis; ρ, Pearson’s correlation coefficient; *P < 0.05; **P < 0.01.

Correlation analysis of the parameters of PDS showed that the number of PDSs and the number of spikes per PDS are significantly correlated (n = 40, Fig. 4A). However, the PDSs appeared to be independent from the ictal-like activity, since no significant correlation was found between the PDS parameters and the ictal duration (Fig. 4B,C). The PDS parameters did not exhibit any significant correlation with τ_50_, τ_90_, or the peak depolarization potential of SD, either (Fig. 4D–I). Taken together, these results indicate that the amplitude and recovery of SD have an influence on the following ictaform activity rather than the later-generated PDSs.

### Influence of AEDs on the electrophysiological properties of the SD waveform

Next, we tested the effects of a range of AEDs on SD parameters and SD-induced epileptiform activity. The tested AEDs include carbamazepine, phenytoin, valproate, lamotrigine, zonisamide, felbamate, gabapentin, levetiracetam, ethosuximide, tiagabine and vigabatrin. The concentration used for each compound was selected as medium to high doses according to previous *in vitro* studies (Table 1). We first examined whether the AEDs tested in our study have a direct modulatory effect on the electrophysiological parameters of SD. Table 2 summarizes τ_50_, τ_90_, and the peak depolarization potential after different AED treatments. Among the 11 tested AEDs, felbamate, tiagabine, and vigabatrin significantly increased τ_50_ of SD, suggesting that these drugs delay the early repolarization phase of SD (Table 2). However, when τ_90_ was measured, only felbamate and vigabatrin caused a significant increase. Treating with the other AEDs did not significantly change the τ_90_ of SD (Table 2). Moreover, phenytoin significantly suppressed the peak depolarization potential, whereas the other AEDs did not influence the extent of depolarization during SD (Table 2).

**Table 1:** Clinical and *in vitro* doses for AEDs.

AED	Dosage in human^63^	Reported doses and mechanisms *in vitro*	Tested doses
Carbamazepine	200–1200 mg/day^a^	Inhibits VGSCs with an IC_50_ of ~30μM^23^	1, 5, and 50 μM
Phenytoin	300–600 mg/day^b^	Inhibits VGSCs with an IC_50_ of ~30 μM^23^	5 and 50 μM
Valproate	10–60 mg/kg/day	Inhibits Na^+^ conductance and changes other parameters of Na^+^ currents at a concentration of 1 mM^24^	1 mM
Lamotrigine	25–375 mg/kg/day^a^	Inhibits human VGSCs with an IC_50_ of 56 μM^26^	60 μM
Zonisamide	100–400 mg/day^b^	Enhances steady-state fast inactivation of Na^+^ current with a dissociation constant of 12 μM and reduces T-type Ca^2+^ currents by 38% at 50 μM^27,64^	50 μM
Felbamate	1200–3600 mg/day^c^	Inhibits neuronal firing in striatal neurons with an IC_50_ of 28 μM and reduces NMDA-mediated currents by 29% at 100 μM^65^	100 μM
Gabapentin	900–3600 mg/day^a^	Inhibits VGCC-mediated currents with an IC_50_ of 167 nM and a saturated dose of 25 μM in DRG neurons^66^	50 μM
Levetiracetam	1000–3000 mg/day^d^	Inhibits VGCC-mediated currents with an IC_50_ of 14.7 μM in hippocampal neurons^67^	100 μM
Ethosuximide	20 mg/kg/day^e^	Inhibits T-type VGCC-mediated currents with an IC_50_ of 500 μM^68^	500 μM
Tiagabine	4–32 mg/day^a^	Prolongs the duration of IPSPs and inhibits epileptiform bursting at a concentration of 25 μM^69^	30 μM
Vigabatrin	0.5–3 g/day^b^	Increased cellular GABA concentrations in slices and tonic GABA currents in cultured neurons at a concentration of 100 μM^70,71^	200 μM

^a^Doses for adults and children over 12 years of age.

^b^Doses for adults.

^c^Doses for adults and children over 14 years of age.

^d^Doses for adults and children over 16 years of age.

^e^The optical dose for pediatric patients.

**Table 2:** Effects of AEDs on parameters of SD.

AED	Dose (μM)	n = (slices)	Ani-mals	τ_50_ (s)	P value	τ_90_ (s)	P value	Peak potential (mV)	P value
Control	—	40	40	26.5 ± 1.1	—	85.0 ± 5.4	—	−3.6 ± 1.0	
Carbamazepine	50	6	4	23.2 ± 1.4	0.59	88.7 ± 14.9	0.99	−6.2 ± 2.3	0.81
Phenytoin	50	6	5	21.2 ± 2.7	0.20	80.7 ± 13.2	0.99	−16.5 ± 5.5	0.0006**
Valproate	1000	6	5	32.7 ± 3.2	0.11	105.8 ± 22.1	0.48	−6.3 ± 3.2	0.79
Lamotrigine	60	5	4	20.4 ± 5.1	0.37	69.1 ± 23.9	0.71	−8.7 ± 2.7	0.33
Zonisamide	50	9	8	27.4 ± 3.0	0.99	79.0 ± 10.1	0.95	−5.6 ± 2.8	0.81
Felbamate	100	8	5	40.6 ± 5.1	0.0003**	121.0 ± 12.4	0.03*	−8.2 ± 3.1	0.26
Gabapentin	50	5	3	28.9 ± 7.1	0.93	66.9 ± 12.1	0.66	−2.0 ± 3.3	0.93
Levetiracetam	100	5	4	23.5 ± 6.9	0.87	76.4 ± 29.2	0.94	−5.4 ± 2.9	0.90
Ethosuximide	500	5	5	26.3 ± 6.1	1.00	59.1 ± 14.0	0.36	−9.6 ± 2.4	0.15
Tiagabine	30	7	4	45.7 ± 4.7	2.0e-6**	87.5 ± 12.1	0.98	−4.8 ± 2.5	0.87
Vigabatrin	200	6	5	38.9 ± 5.3	0.003**	131.0 ± 24.7	0.01*	−5.8 ± 1.8	0.67

Data are presented as mean ± S.E.M. Significance differences were compared between the control group and the AED groups and were determined by a one-way ANOVA followed by two-tailed Dunnett-t post hoc test. *p < 0.05; **p < 0.01.

### The anti-epileptic effects of older generation of AEDs that influence Na^+^ channels

**Figure 5:**
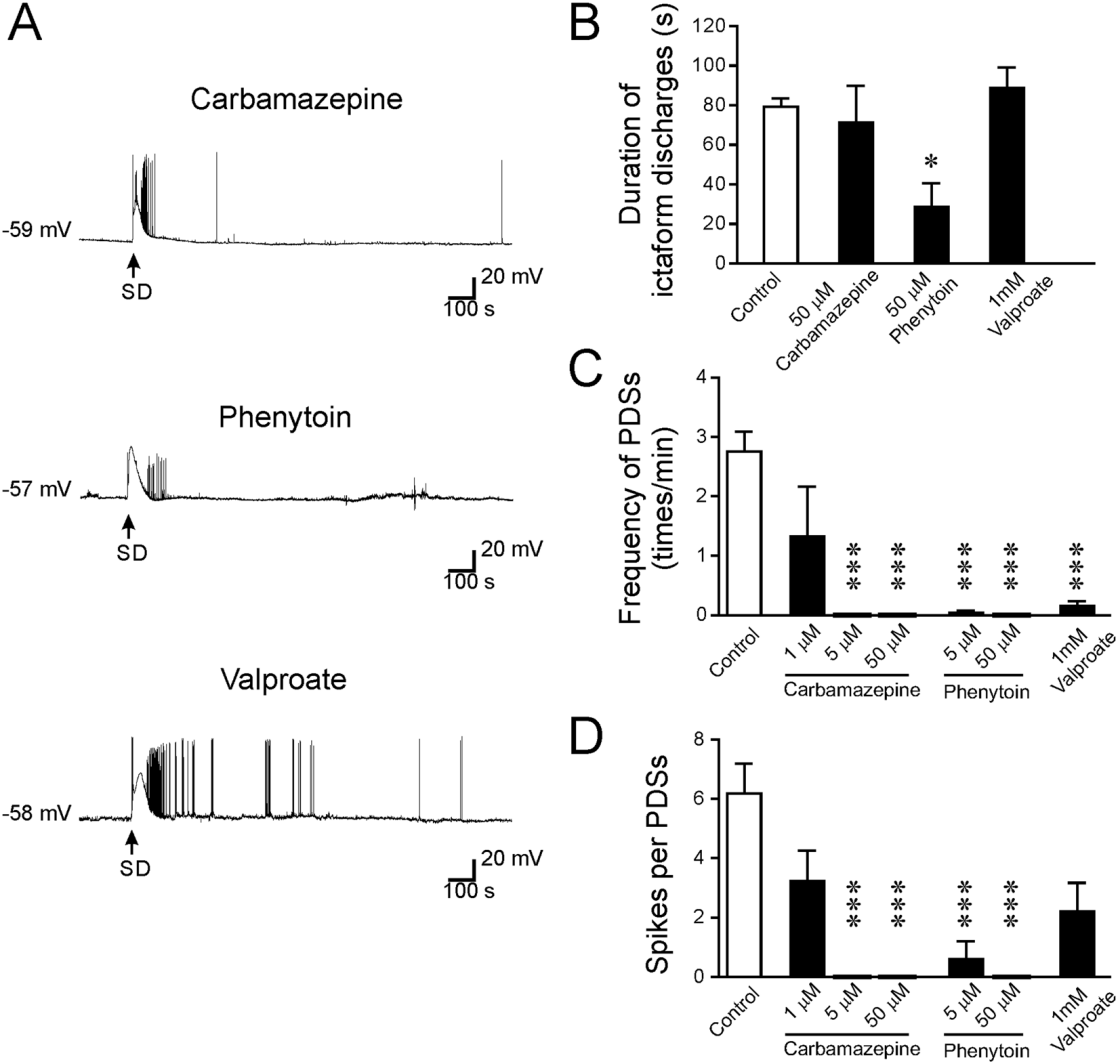
The effects of carbamazepine, phenytoin, and valproate on SD-induced epileptiform activity. (**A**) Representative traces showing the changes in the membrane potential after treatment with carbamazepine (50 μM), phenytoin (50 μM), or valproate (1 mM). (**B**) Quantitative results showing the effects of AEDs on the duration of ictal-like activity. (**C**) Quantitative results showing the frequency of PDSs after treatment with carbamazepine (1–50 μM), phenytoin (5-50 μM), or valproate (1 mM) than in control. (**D**) Quantitative results showing the number of spikes per PDS after treatment with carbamazepine (1–50 μM), phenytoin (5–50 μM), or valproate (1 mM) than in control. Significance differences were determined by a One-way ANOVA with the Games-Howell post hoc test and were defined as *P < 0.05; **P < 0.01; ***P < 0.001.

The first generation of AEDs are widely used in clinic due to their well-documented pharmacokinetics profiles. Among these drugs, carbamazepine, phenytoin, and valproate are known to prevent epilepsy by inhibition of VGSC activity^22–25^. We first tested the effects of high concentrations of the three AEDs on SD-induced ictal duration and PDS parameters (Fig. 5). Our results show that 50 μM phenytoin significantly reduced the duration of ictaform discharges that occurred at the late repolarization phase of SD (28.7 ± 11.9 s, n = 6, p = <0.05 compared with control: 79.2 ± 4.3 s, n = 40), whereas carbamazepine (50 μM, 71.3 ± 18.7 s, n = 6, p = 0.97) or valproate (1 mM, 88.7 ± 10.3 s, n = 6, p = 0.83) did not show a significant effect. Next, we examined the effects of these AEDs on SD-induced PDSs. The three tested AEDs at high concentrations all potently inhibited the induction of PDSs after SD. There were no PDSs after treatment with carbamazepine (n = 6) or phenytoin (n = 6). Valproate strongly decreased frequency of PDSs (0.16 ± 0.07 times/min, n = 5, p < 0.001) and spike number per PDS (2.2 ± 1.0, n = 5, p = 0.13). Since high doses of carbamazepine and phenytoin showed a complete block on PDSs, we further tested if the lower concentrations of these AEDs were also effective. Our data showed that carbamazepine still completely blocked the generation of PDS after SD at a concentration of 5 μM, but failed to produce a significant effect at 1 μM (1.3 ± 0.8 PDS/min for the frequency of PDSs, p = 0.70; and 3.2 ± 1.0 for the number of spikes/PDS, p = 0.43; n = 5). Phenytoin at a lower concentration of 5 μM still significantly inhibited the generation of PDSs after SD (0.04 ± 0.04 times/min for the frequency of PDSs, p < 0.001; and 0.6 ± 0.6 for the number of spikes/PDS, p < 0.001; n = 5). Taken together, these results indicate that carbamazepine, phenytoin, and valproate are effective in suppressing the SD-induced epileptiform activity.

### The anti-epileptic effects of newer generation of AEDs that possibly influence Na^+^ channels

**Figure 6:**
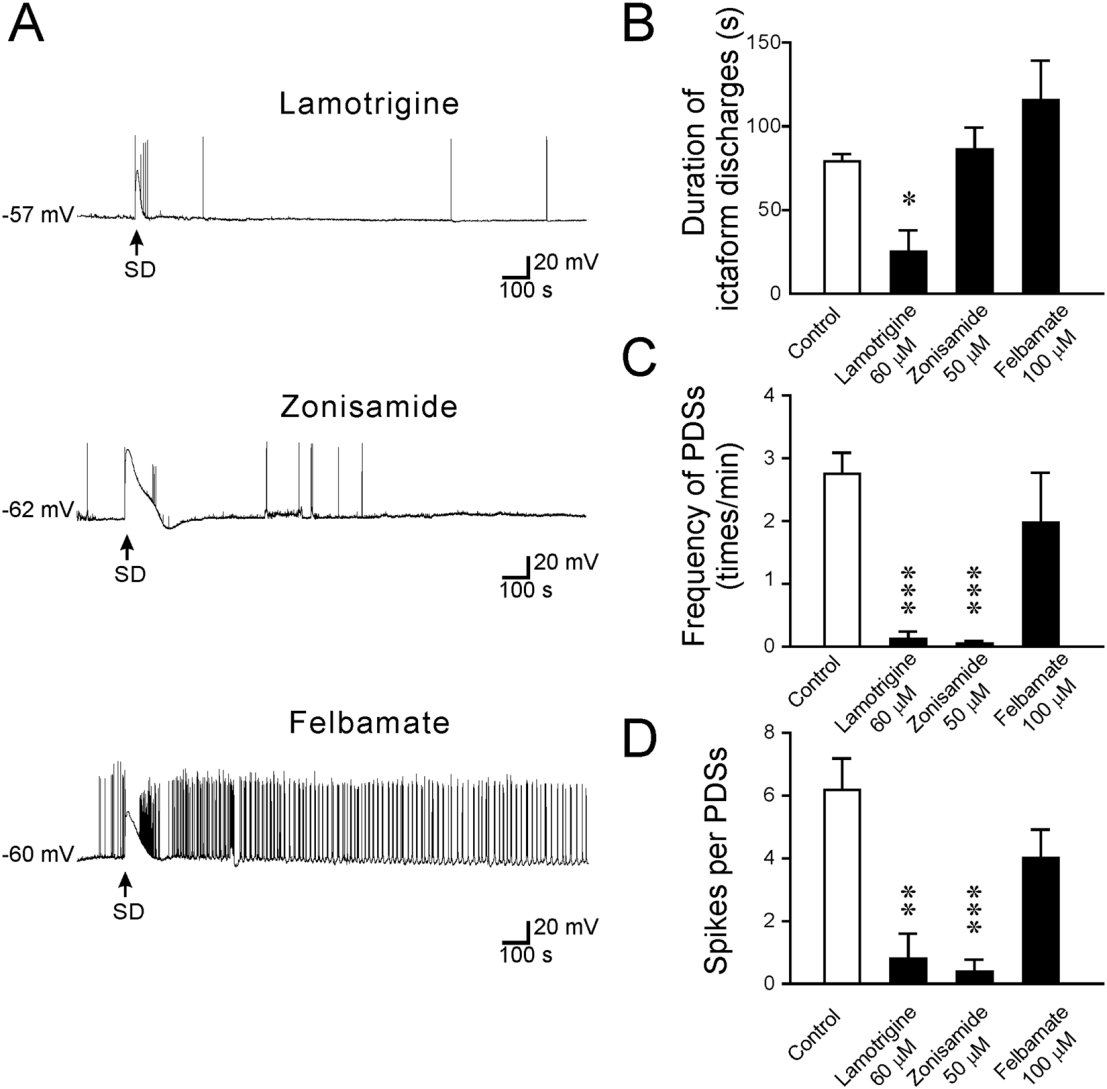
The effects of lamotrigine, zonisamide, and felbamate on SD-induced epileptiform activity. (**A**) Representative traces showing the changes in the membrane potential after treatment with lamotrigine (60 μM) and zonisamide (50 μM) or felbamate (100 μM). (**B**) Quantitative results showing the effects of AEDs on the duration of ictal-like activity. (**C**) Quantitative results showing the frequency of PDSs after treatment with lamotrigine (60 μM) and zonisamide (50 μM), or felbamate (100 μM) compared to that of control. (**D**) Quantitative results showing the number of spikes per PDS after treatment with lamotrigine (60 μM) and zonisamide (50 μM) or felbamate (100 μM) compared to control. Significance differences were determined by a One-way ANOVA with the Games-Howell post hoc test and were defined as *P < 0.05; **P < 0.01; ***P < 0.001.

The newer generation of AEDs has shown improved tolerability and safety. Among these drugs, lamotrigine, zonisamide, and felbamate have been reported to modulate VGSC-mediated neuronal responses^26–28^. Figure 6 summarizes the effects of these drugs on SD-induced epileptiform activity. Among the tested AEDs, only lamotrigine (60 μM; 25.1 ± 12.8 s, n = 5, p < 0.05) significantly inhibited the duration of SD-induced ictal-like discharges, whereas zonisamide (50 μM; 86.3 ± 13.0 s, n = 9, p = 0.95) or felbamate (100 μM; 115.7 ± 23.6 s, n = 8, p = 0.47) did not show a significant effect. Both lamotrigine and zonisamide significantly reduced the frequency of PDS generation after SD (0.12 ± 0.12, n = 5, p < 0.001 after treatment with lamotrigine; 0.04 ± 0.04, n = 9, p < 0.001 after treatment with zonisamide). The number of discharges during each PDS was also decreased after treatment with lamotrigine (0.8 ± 0.8, n = 5, p < 0.01) or zonisamide (0.4 ± 0.4, n = 9, p < 0.001). The treatment with 100 μM felbamate failed to influence the frequency of the PDSs (1.98 ± 0.80, n = 8, p = 0.81) or the spike numbers during the PDSs (4.01 ± 0.90, n = 8, p = 0.39). These data indicate that both lamotrigine and zonisamide effectively attenuated the SD-induced generation of PDSs, whereas felbamate had no significant effect.

### Effects of AEDs that affects VGCCs

**Figure 7:**
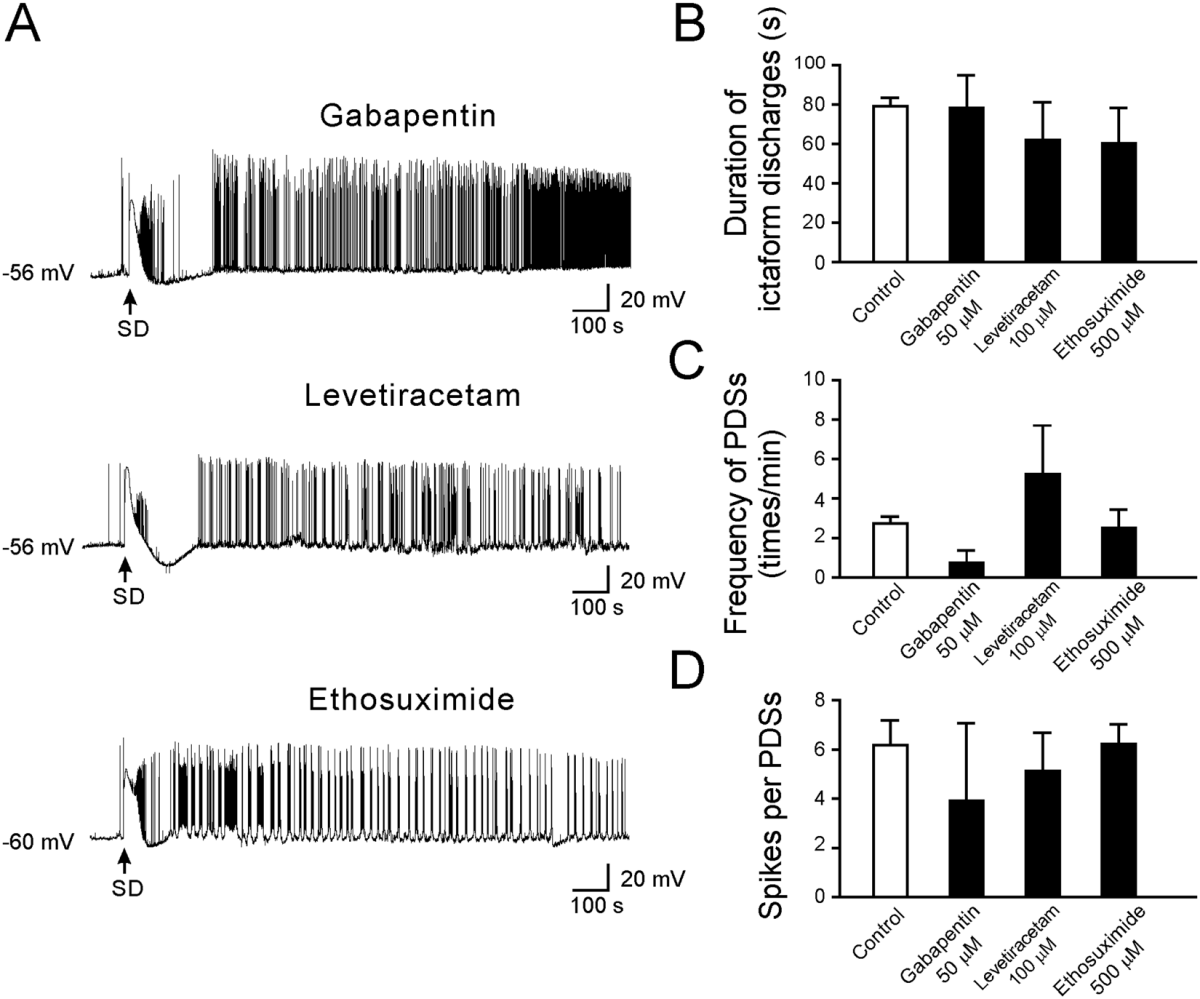
The effects of gabapentin, levetiracetam, and ethosuximide on SD-induced epileptiform activity. (**A**) Representative traces showing the changes in the membrane potential after treatment with gabapentin (50 μM), levetiracetam (100 μM), and ethosuximide (500 μM). (**B**) Quantitative results showing the effects of AEDs on the duration of ictal-like activity. (**C**) Quantitative results showing the frequency of PDSs after treatment with gabapentin (50 μM), levetiracetam (100 μM), and ethosuximide (500 μM) compared to that of control. (**D**) Quantitative results showing the number of spikes per PDS after treatment with gabapentin (50 μM), levetiracetam (100 μM), and ethosuximide (500 μM) compared to that of control. Significance differences were determined by a One-way ANOVA with the Games-Howell post hoc test and were defined as *P < 0.05; **P < 0.01; ***P < 0.001.

Gabapentin, levetiracetam, and ethosuximide are AEDs that are thought to mainly inhibit VGCCs. Gabapentin and levetiracetam mainly target the high-voltage activated Ca^2+^ channels^29,30^, while ethosuximide is a specific drug for absent seizures that works by blocking T-type VGCCs in the thalamus^31^. The duration of ictaform discharges immediately following SD was not affected by gabapentin (50 μM; 78.3 ± 16.6 s, n = 5, p = 1.00), levetiracetam (100 μM; 62.2 ± 19.0 s, n = 5, p = 0.82), or ethosuximide (500 μM; 60.3 ± 18.1 s, n = 5, p = 0.75). The frequency of PDSs was 0.76 ± 0.62 time/min (n = 5, p = 0.10) after treatment with gabapentin, 5.24 ± 2.47 time/min (n = 5, p = 0.76) after levetiracetam, and 2.52 ± 0.94 time/min (n = 5, p = 0.99) after ethosuximide. The number of spikes per PDS was 3.92 ± 3.15 (n = 5, p = 0.90) after treatment with gabapentin, 5.13 ± 1.55 (n = 5, p = 0.94) after levetiracetam, and 6.22 ± 0.80 (n = 5, p = 1.00) after ethosuximide. Therefore, none of these AEDs induced a significant inhibition on either the ictaform activity or the PDSs after SD (Fig. 7). These data suggest that SD-induced epileptiform activity is not sensitive to AEDs that modulate Ca^2+^ currents.

### Effects of AEDs that modulate GABAergic transmission

**Figure 8:**
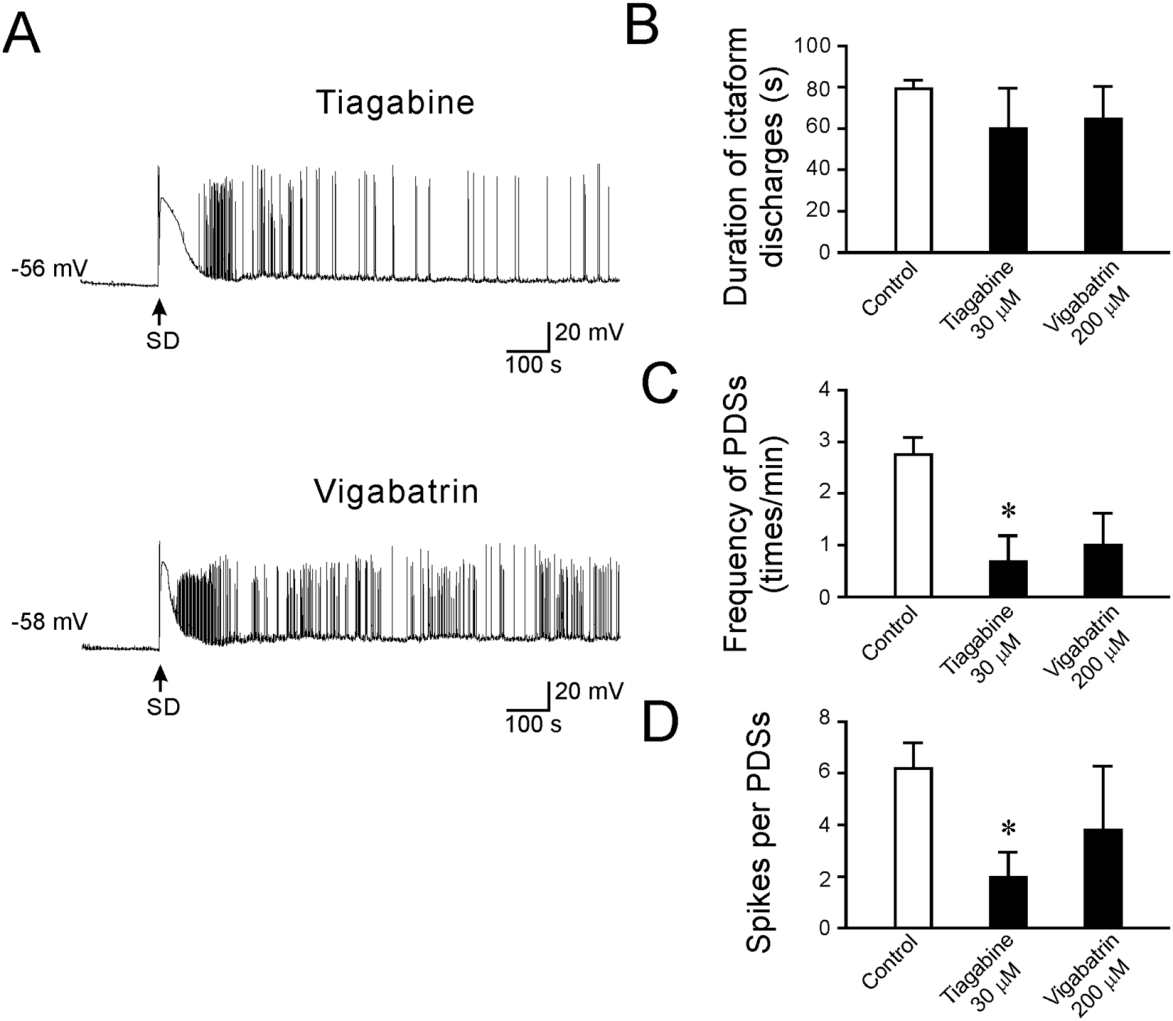
The effects of tiagabine and vigabatrin on SD-induced epileptiform activity. (**A**) Representative traces showing the changes in the membrane potential after treatment with tiagabine (30 μM) and vigabatrin (200 μM). (**B**) Quantitative results showing the effects of AEDs on the duration of ictal-like activity. (**C**) Quantitative results showing the frequency of PDSs after treatment with tiagabine (30 μM) and vigabatrin (200 μM) compared to that of control. (**D**) Quantitative results showing the number spikes per PDS after treatment with tiagabine (30 μM) and vigabatrin (200 μM) compared to that of control. Significance differences were determined by a One-way ANOVA with the Games-Howell post hoc test and were defined as *P < 0.05; **P < 0.01; ***P < 0.001.

The anticonvulsant actions of tiagabine and vigabatrin are mainly through the potentiation of GABAergic signaling. Tiagabine potentiates GABA-mediated synaptic responses by inhibiting GABA uptake transporters^32^. Vigabatrin is a GABA analogue that irreversibly inhibits GABA transaminase and increases the extracellular GABA concentration and tonic inhibition^33^. Our results showed that the duration of ictaform activity was not affected by tiagabine (30 μM; 59.9 ± 19.6 s, n = 7, p = 0.63) or vigabatrin (200 μM, 64.5 ± 15.9 s, n = 6, p = 0.67). Tiagabine significantly reduced the number of PDSs (0.69 ± 0.50 time/min, n = 7, p < 0.05) and the spike number per PDS (1.98 ± 0.96, n = 7, p < 0.05). Vigabatrin produced a reducing trend on the frequency of PDSs (1.00 ± 0.62 time/min, n = 7); however, no statistical significant was reached for either PDS frequency (p = 0.08) or the number of spikes per PDS (3.80 ± 2.49, n = 7, p = 0.66) (Fig. 8). These results suggest that tiagabine rather than vigabatrin has an inhibitory effect on SD-induced PSDs.

## Discussion

SD and epilepsy are both episodic disorders caused by brain hyperexcitation or damage. They share many clinical features as well as some underlying mechanisms^34^ and could co-occur in the cerebral cortex^7,35^. In rat hippocampal slices, both SD and epilepsy could be induced by repetitive stimulation, especially under conditions of low extracellular Mg^2+^ concentrations^36^. In human slices, SD enhanced the repetition rate and amplitude of spontaneous seizure-like activity^37^. Instances of both SD and epilepsy implicated the role of ionotropic glutamate receptors. Moreover, the results from clinical studies suggest that the occurrence of seizures is highly associated with SD after acute brain injury^38^. The ictal epileptic field potentials in particularly could start at the end of the potential change of SD in aSAH patients^6^. Epilepsy is a well-recognized complication that occurs after acute brain injuries, especially including hemorrhagic stroke and TBI^39,40^. Many types of AEDs have been used as prophylaxis to prevent the onset of seizures and late seizures after brain injury conditions^41^. For example, phenytoin, carbamazepine and valproate are traditionally used as the primary choice of prophylactic drugs after aSAH; newer AEDs, such as levetiracetam, lamotrigine, gabapentin, and zonisamide, have also been used because of their better side effect profiles^39^. However, the effects of these AEDs are uncertain due to the lack of randomized and placebo controlled trials. The mechanisms of epileptogenesis after brain injury also remain largely unclear. Some studies have demonstrated that one contributing factor is the disruption of the blood-brain barrier after these conditions^42^. Functional changes in astrocytes could also enhance hyperexcitability and hyper-synchronization among neurons^43^. The alterations in the blood-brain barrier and glial cells could be further aggravated by epilepsy and SD^44–46^. Moreover, electrocorticography (ECoG) recordings showing that SD precedes ictal discharges in some aSAH patients suggest that SD could directly or indirectly increase the susceptibility to seizures under the condition of brain damage^6^. Effective attenuation of epileptiform activity after SD may help to reduce seizures in these patients.

In the present study, we showed that epileptiform activity could be induced by SD under a condition of increased neuronal excitability, since internal TEA could enhance EPSP, increase membrane input resistance, and produce a slight membrane depolarization^47,48^. Under these conditions, SD first induced a phase of ictaform events that occurred at the late repolarization phase. The duration of the ictaform discharges are significantly correlated with τ_50_, τ_90_, and the peak potentiation potential of SD, suggesting that a longer recovery time and a more depolarized state of SD favors the occurrence of ictal-like activity. In the present model, SD also induced a prolonged phase of interictal-like activity, which was shown as the PDSs. SD-induced PDSs have also been observed in previous studies, in which PDS persisted for ~1 h in rat neocortical and hippocampal slices under a condition of 1.25 µM bicuculline that leads to partial disinhibition. However, the mechanism for the generation of PDSs remains unknown. Our results showed that the parameters of PDSs, including the frequency and spike numbers per PDS, were not significantly correlated with the parameters of SD or the parameter of ictaform activity. It is possible that the SD-triggered strong ionic and metabolic responses disrupt the balance between neural excitation and inhibition. Under a condition of partial disinhibition, PDSs could be evoked when SD nudges this balance towards hyper-excitation.

Carbamazepine, phenytoin, lamotrigine, valproate, and zonisamide are potent VGSC inhibitors. These AEDs block high-frequency repetitive spike discharges without significantly affecting physiological action potentials^12,49^. In this study, carbamazepine, phenytoin, lamotrigine, valproate, and zonisamide reduced not only the number of overriding action potentials during the PDS but also the incidence of PDSs after SD. This could be due to multiple mechanisms of actions of these AEDs. First, VGSC-targeting AEDs, such as phenytoin and lamotrigine, produce a tonic inhibition on VGSCs by voltage-dependent and use-dependent manners^22,50^. They preferentially bind to the inactivated conformation of the channel and exhibited stronger inhibition when the depolarization state was prolonged and accumulated^26,49^. During the PDSs, the prolonged depolarization of the neuronal membrane causes repetitive discharges, which are sensitive to AED blockade^11^. Second, the binding and unbinding kinetics of these AEDs are slow^51,52^. In our experiments, the onset of SD is accompanied by a sustained depolarization of the membrane potential that reaches ~0 mV and lasts for tens of seconds. Such a condition already favors the binding of AEDs and therefore prevents the generation of PDSs. Third, some AEDs might also inhibit the persistent Na^+^ currents. Persistent Na^+^ currents contribute to the initiation and enhancement of PDSs when the PDS is induced by an EPSP^11,53^. Therefore, inhibition of persistent Na^+^ currents could reduce the extent of depolarization during the PDS. Together, these properties of VGSC-targeting AEDs might contribute to the suppression of both the incidence of PDSs and the overriding discharges induced by SD.

We also tested VGCC-targeting AEDs including gabapentin, levetiracetam, and ethosuximide, for their ability to affect epileptiform activity after SD. Blocking Ca^2+^ channels could inhibit the release of neurotransmitters and attenuate postsynaptic excitability^54^. Gabapentin and levetiracetam are thought to modulate high voltage-activated Ca^2+^ channels, whereas ethosuximide mainly inhibits T-type Ca^2+^ channels. However, the pharmacological mechanisms of these AEDs are complex. For example, levetiracetam is known to modulate N-type Ca^2+^ channels^30^, and it also binds to the synaptic vesicle protein SV2A to modulate neurotransmitter release and stabilizes GABA_A_ receptors to facilitate inhibition^55,56^. In our study, gabapentin, levetiracetam, and ethosuximide did not show significant inhibition of the generation of PDSs, suggesting that SD-induced epileptiform activity was relatively insensitive to this type of AED. In a previous study, however, levetiracetam could reduce the duration and depolarization magnitude of PDSs induced by Mg^2+^-free solutions containing 4-aminopyridine and bicuculline^57^. This could largely result from the difference in the condition of the induction of epilepsy: in the study by Pisani *et al*., GABAergic transmission was blocked and NMDA receptors were potentiated. Such a condition could be more favorable to the generation of PDSs that are initiated by the largely enhanced excitatory synaptic transmission^10^. The increased synaptic release and the resulting PDSs therefore largely depend on activation of VGCCs and are sensitive to levetiracetam.

The GABAergic transmission is also involved the SD-induced epileptiform activity. The slices are more susceptible to generate epilepsy after SD when low concentrations of GABA_A_ receptor antagonists were applied^8^. It has been reported that SD causes changes in receptor binding sites of both glutamate and GABA receptors and leads to an imbalance between excitatory and inhibitory inputs during the late phase^58,59^. The spontaneous and miniature inhibitory postsynaptic currents exhibited decreased frequency and increased amplitude after SD^60^. Therefore, the altered GABAergic transmission might contribute to epileptogenesis after SD. In this study, we show that the frequency of PDSs induced by SD was reduced by tiagabine. Tiagabine is a potent and selective inhibitor of the GABA uptake transporter GAT1^32^, which is responsible for removing GABA from the synaptic cleft. Tiagabine can slow the reuptake of GABA from synapses and prolong the inhibitory postsynaptic potential. Our results suggest that enhancing GABA inhibition might attenuate SD-induced epileptiform activity.

In conclusion, our study reveals that AEDs that mainly target VGSCs, including carbamazepine, phenytoin, valproate, lamotrigine, and zonisamide, are potent inhibitors of SD-induced epileptiform activity. Tiagabine, which mainly modulates GABAergic transmission, also exhibits moderate inhibitory effects. AEDs that inhibit VGCCs, including gabapentin, levetiracetam, and ethosuximide, do not significantly affect the generation of PDSs after SD. These data will have implications for the therapy of epilepsy as a complication of SD-related neurological disorders.

## Methods

### Preparation of brain slices

All experimental procedures were in accordance with the Institutional Guidelines of China Medical University for the Care and Use of Experimental Animals (IGCMU-CUEA) and were approved by the Institutional Animal Care and Use Committee (IACUC) of China Medical University. Hippocampal slices were prepared from 12 to 21-day-old ICR mice of either sex. Mice were anaesthetized with urethane and decapitated. Brains were removed and placed in ice-cold artificial cerebrospinal fluid (ACSF) containing the following (in mM): 126 NaCl, 2.5 KCl, 2.0 MgCl_2_, 2.0 CaCl_2_, 1.25 NaH_2_PO_4_, 26 NaHCO_3_, and 10 D-glucose. Then, 350 μm-thick transverse hemi-sections from the hippocampus were sliced (Leica vibratome). The slices were incubated at room temperature for >1 h before recording. The slices were then transferred to the recording chamber with fresh ACSF containing the following (in mM): 125 NaCl, 5.0 KCl, 1.3 MgCl_2_, 2.0 CaCl_2_, 1.25 NaH_2_PO_4_, 26 NaHCO_3_, and 10 D-glucose. All solutions were saturated with 95% O_2_/5% CO_2_. In all pharmacological experiments, slices were pre-treated with chemicals for 20 min before SD induction unless specifically mentioned otherwise.

### Whole-cell patch clamp recordings

Whole-cell patch clamp recordings were performed in CA1 pyramidal neurons using a MultiClamp 700B amplifier. The membrane potential was acquired under current-clamp mode without any holding current applied (I = 0 mode). Patch electrodes (3–7 MΩ) were pulled from 1.5-mm outer diameter thin-walled glass capillaries in three stages and were filled with intracellular solutions containing the following (in mM): 98 K-gluconate, 17 KCl, 10 HEPES, 1.1 EGTA, 0.1 CaCl_2_, 25 TEA-Cl, and 2 Na_2_-ATP, pH 7.25, osmolarity 290–300. Input resistance was obtained before and after each recording and recordings with a >25% change in input resistance were discarded. Signals were acquired via a Digidata 1440 A analog-to-digital interface and were low-pass filtered at 2 kHz and digitized at 10 kHz.

### Induction and acquisition of SD and SD-associated epileptiform activity

Individual slices were transferred to a recording chamber (Fast Exchange Diamond Bath Chamber from Warner Instruments, Hamden, CT, USA) perfused with oxygenated ACSF at room temperature. SD was induced with a brief focal ejection of 2.5 M KCl from a glass pipette (resistance ~1–3 MΩ). The puffing pipette was placed close to the surface of the slice (z < 5 μm) in the CA3 stratum radiatum. Whole-cell recordings were made from CA1 pyramidal neurons at locations >200 μm from the KCl ejection site. One neuron was recorded in each slice, and a fresh slice was used for each new trial. The onset of SD was indicated by the well-established electrophysiological criteria for an SD: abruptly developing depolarization of neuronal membrane potential to nearly 0 mV^61^. Under a condition of 1.3 mM extracellular Mg^2+^, 5.0 mM extracellular K^+^ and intracellular TEA that helps to enhance excitability of the recorded neuron, intensive ictal-like activity occur in the repolarizing phase of SD. The peak depolarization potential was measured as the highest value of the absolute membrane potential reached during the SD. The half-width duration of the SD was measured at the half-maximum of the potential shifts. τ_50_ and τ_90_ were determined by measuring the time intervals from the peak depolarization potential to the point when membrane potential returns to 50% and 90% of resting membrane potentials, respectively. The duration of ictal-like activity was measured by the duration of continuous bursts (1~5 Hz) that occurred during the repolarization phase of SD. PDSs normally appeared after the ictal-like activity had stopped and were identified by a prolonged depolarization plateau with overriding repetitive discharges (>=3) with a frequency of >=5 Hz. Depolarization shifts with 2 spikes (and less) may represent the physiological firing pattern of “early-bursting pyramidal neurons” and thereby were not considered as PDSs^62^. The frequency of PDS was calculated as times/min. The spike number was calculated as the number of repetitive spikes during each PDS. If there was no PDS occurred, the frequency of PDS and the spike number/PDS were both considered 0. The frequency of PDS and spike number were analyzed as a mean value each min during the 20–25 min after the onset of SD in Figs 5–8.

### Chemicals

Phenytoin, levetiracetam, ethosuximide, and tiagabine were obtained from Sigma-Aldrich (St. Louis, MO, USA). Carbamazepine, valproate, lamotrigine, zonisamide, felbamate, gabapentin, and vigabatrin were obtained from Tocris (Avonmouth, Bristol, UK).

### Data analysis

Data in all figures are reported as the mean ± standard error of mean (s.e.m.). Statistical analysis was performed using the Statistical Product and Service Solutions (SPSS, IBM). One-way analysis of variance with the Games-Howell post hoc test or the two-tailed Dunnett-t post hoc test was used for statistical comparisons of multiple groups. Pearson’s correlation coefficient (ρ) was used for correlation analysis. Statistical significance was defined as *p < 0.05; **p < 0.01; ***P < 0.001.
